# A user-friendly Matlab program and GUI for the pseudorotation analysis of saturated five-membered ring systems based on scalar coupling constants

**DOI:** 10.1186/1752-153X-2-20

**Published:** 2008-10-24

**Authors:** Pieter MS Hendrickx, José C Martins

**Affiliations:** 1 NMR and Structure Analysis Unit, Ghent University, Krijgslaan 281 S4, B-9000 Ghent, Belgium

## Abstract

**Background:**

The advent of combinatorial chemistry has revived the interest in five-membered heterocyclic rings as scaffolds in pharmaceutical research. They are also the target of modifications in nucleic acid chemistry. Hence, the characterization of their conformational features is of considerable interest. This can be accomplished from the analysis of the ^3^*J*_*HH *_scalar coupling constants.

**Results:**

A freely available program including an easy-to-use graphical user interface (GUI) has been developed for the calculation of five-membered ring conformations from scalar coupling constant data. A variety of operational modes and parameterizations can be selected by the user, and the coupling constants and electronegativity parameters can be defined interactively. Furthermore, the possibility of generating high-quality graphical output of the conformational space accessible to the molecule under study facilitates the interpretation of the results. These features are illustrated via the conformational analysis of two 4'-thio-2'-deoxynucleoside analogs. Results are discussed and compared with those obtained using the original PSEUROT program.

**Conclusion:**

A user-friendly Matlab interface has been developed and tested. This should considerably improve the accessibility of this kind of calculations to the chemical community.

## Background

Five-membered heterocyclic ring systems constitute an important part of many biologically relevant molecules. They occur in carbohydrates (furanoses), nucleosides and nucleotides, the amino acid proline and their many derivatives. In addition, they often occur as a moiety in complex natural products. Chemical modifications of nucleic acids, often driven by the needs of antisense research, target in part the five-membered cycle or its analogues in order to tailor their conformation towards the desired needs [1,2].

The advent of combinatorial chemistry has also revived the interest in heterocyclic rings and their conformation [3]. As a result, scaffolds containing five-membered heterocycles have received much attention for the rapid generation of potential lead compounds in pharmaceutical research [4-11].

Typically, the chemical and conformational space is explored by introducing a diversity of substituents at varying positions around the cycle. Depending on the position and nature of these substituents, the cycle either adopts a single conformation or may be in equilibrium between two conformations. These conformations will in turn impact on the conformational space that will be covered by the substituents, making the determination of the cycle's conformation an issue of considerable interest.

Over the years, NMR has become a well-established technique for this purpose. In particular, ^3^*J*_*HH *_scalar coupling constants are well-suited as they are mainly determined by the torsion angle over which they are measured. In the case of ring systems, the vicinal ^3^*J*_*HH *_scalar coupling constants are directly correlated to their corresponding exocyclic torsion angles (*θ*_*exo*_). These are related to the corresponding endocyclic torsion angles (*θ*_*endo*_) by a simple equation (1) where *A *and *B *are constants determined by the geometry of the atoms linked to the common central bond.

(1) *θ*_*exo *_= *A θ*_*endo *_+ *B*

As the set of all five endocyclic torsion angles in a five-membered ring fully determines its conformation, ^3^*J*_*HH *_scalar coupling constants provide a direct measure of the ring's conformation. The Haasnoot-Altona equation (3) [12] and the Diez-Donders equation (4) [13,14], both based on the well known Karplus equation (2), describe the relation between a ^3^*J*_*HH *_coupling and the corresponding exocyclic torsion angle (*θ*_*exo*_) to a high level of accuracy. In both equations this is mainly achieved by including a set of four parameters *λ*_*i *_(*i *= 1, ..., 4) that account for the influence of electronic effects contributed by the substituents [15,16]. In some studies, the set of experimental ^3^*J*_*HH *_scalar coupling constants is further extended by ^3^*J*_*HF *_scalar coupling constants [17-19] or interproton distances obtained by nOe NMR experiments. Here however, we assume that only ^3^*J*_*HH *_scalar couplings are available for conformational analysis.

(2) ^3^*J*_*HH *_= *P*_1 _*cos*^2^(*θ*) + *P*_2 _*cos*(*θ*) + *P*_3_

(3) $$\begin{array}{c} {\;}^{3}J_{HH}=P_{1}\;cos^{2}(\theta )+P_{2}\;cos(\theta )+P_{3}+\\ \sum_{i=1}^{4}\lambda_{i}\left\{P_{4}+P_{5}\;cos^{2}({\left(-1\right)}^{i}\lambda_{i}\theta +P_{6}\left|\lambda_{i}\right|)\right\} \end{array}$$

(4) $${\;}^{3}J_{HH}=\sum_{i=0}^{3}C_{i}(\lambda_{1\ldots 4})\;cos(i\theta )+\sum_{i=1}^{3}S_{i}(\lambda_{1\ldots 4})\;sin(i\theta )$$

Altona and Sundaralingam showed that the description of a five-membered ring conformation can be reduced to a two-parameter pseudorotation model [20,21] that fully describes its conformation. The first parameter, the pucker phase *P*, represents the phase of the conformation and indicates which ring atoms are positioned out of the ring plane. The second parameter, the pucker amplitude *ν*_*max*_, corresponds to the amplitude of the conformation and describes the extent to which the atoms determined by *P *are out of the plane. The relationship with the endocyclic torsion angles *θ*_*endo*, *i *_is shown in (5).

(5) $$\theta_{endo,i}=\nu_{max}\;cos(P+\frac{4\pi i}{5})$$

This well-known pseudorotation description, originally described for the furanose ring in nucleosides and nucleotides [20,21], was further generalized to any five-membered heterocycle by Diez et al. [22-24] who introduced two additional parameters *α*_*i *_and *ε*_*i *_for each endocyclic bond to cope with differences in bond lengths in various types of five-membered rings (Equation 6). As the phase of the conformation *P *is a periodic variable, polar plots called pseudorotation wheels are mostly used to depict ring conformations.

(6) $$\theta_{endo,i}=\alpha_{i}\nu_{max}\;cos(P+\epsilon_{i}+\frac{4\pi i}{5})$$

Using the above equations, ^3^*J*_*HH *_couplings can be used to derive the pseudorotation parameters of the five-membered cycle. As mentioned previously, the cycle may be in equilibrium between two conformations. Thus, most generally two sets of pseudorotation parameters (*P *and *ν*_*max*_) and the relative population (%_1_, i.e. the percentage of the first conformation present with %_2 _= 1 - %_1_) need to be fitted to the experimental NMR data. In order to avoid an under-determined model, experimental data measured at different temperatures is generally used. In such cases, the model assumes that only the relative population of the two conformations varies when changing the temperature. Thus *n *+ 4 (*n *being the number of temperatures used) variables will be optimized to fit the experimental data in such cases. To the best of our knowledge, the program PSEUROT [25], originally developed by Altona et al., is still the only generally available program to perform this type of analysis. Written in FORTRAN, its interface as well as its output is purely text-based. In order to facilitate the analysis of the PSEUROT results, a post-processing feature has been included in the independently developed MULDER package [26] to generate a graphical output of the PSEUROT results. In this communication, we propose an integrated, user-friendly Matlab program, including a self-explanatory graphical user interface (GUI), to facilitate the set-up, execution and subsequent analysis of pseudorotation calculations for five-membered ring systems decorated with a variety of substituents. The use of Matlab as high-level programming language enables to create, within a limited time frame, high-quality plots that provide a graphical impression of the conformational space accessible. Furthermore, due to the open-source GNU GPL license, users have the opportunity to adapt the program to their specific needs.

## Implementation

**Figure 1:**
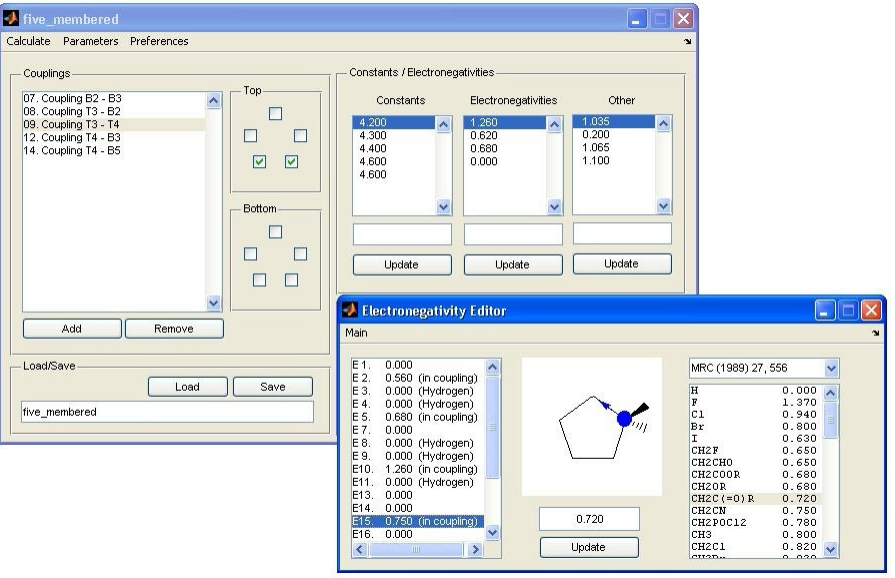
Layout of the Matlab GUI. In the main window of the GUI (upper panel), both the coupling data and additional parameters required by equations 1 and 6 can be interactively defined, using a simplified representation of the cycle. Both the atom types in the cycle and the substituents can be defined interactively. The 'electronegativity editor' (bottom panel) provides a convenient graphical aid to set up the group-electronegativities (*λ*_*i*_) for many common substituents as required by equation 4.

The program consists of a computational core that is accessed and controlled through a GUI (Figure 1), both written in Matlab. Its goal is to search pseudorotation parameters {*P*, *ν*_*max*_} for at most two conformations as well as their relative population (%_1, *n*_) at *n *temperatures that fit a series of experimental NMR scalar coupling constants. The initial pseudorotation parameters are set by the user at the start of the computational procedure. The user is also given the choice to define a subset of pseudorotation parameters that have to be optimized. Using equations 1, 4 and 6, the scalar coupling constants relating to conformation defined by the initial pseudorotation values are calculated and a root-mean-square-deviation (RMSD) is determined with respect to the experimental coupling data. Next, the pseudorotation parameters are adapted so as to minimize this RMSD. The Matlab *fmincon *function from the Optimization Toolbox, which is based on a Sequential Quadratic Programming (SQP) algorithm [27], was used for this purpose. To restrict the optimization to physically sensible solutions, values of partition coefficients were restrained to the [0, 1] interval. Furthermore, puckering amplitudes (*ν*_*max*_) were restricted to the physically relevant [0, *π*/3] interval.

A choice is provided between two operational modes. In the first mode, an optimization of the chosen parameters is performed as is, yielding the pseudorotation parameters that best fit the experimental data. The output generated in this operation mode is purely text-based and contains the optimized variables, their corresponding endocyclic torsion angles and a tabular comparison between experimental and fitted scalar coupling data (See accompanying manual). In the second mode of the program, the complete pseudorotation space of the cycle of interest is explored via 3600 combinations of pseudorotation parameters {*P*, *ν*_*max*_} as described hereafter. The pseudorotation space of the cycle is covered by sampling 120 values of *P *in the [0, 2*π*] interval and 30 values of *ν*_*max *_in the [0, *π*/3] interval. For each {*P*, *ν*_*max*_} thus sampled, the minimized RMSD between the experimental and calculated scalar couplings is determined. As for the output, a pseudoratation wheel is plotted and the RMSD is represented through 20 contour lines in the [Δ_*min*_, 2Δ_*min *_+ 0.1] interval where Δ_*min *_is the lowest RMSD obtained over the 3600 fittings.

Whether the parameters of a second conformation and the relative populations at each temperature are optimized during each of the 3600 runs is again determined by the user's choice. This results in two possible outputs for this operating mode. For the case where the user chooses not to optimize any parameters of the second conformation, one can easily assess from the pseudoratational wheel if the experimental data can be fitted by a single five-membered ring conformation. For the case where the second conformation's parameters (and the relative population of both conformations) are also to be optimized, the pseudorotation wheel represents the conformational space accessible to the cycle when two five-membered ring conformations are fit to the experimental data. In this latter case, one additional pseudorotation wheel is created for each temperature depicting the relative population of the two fitted conformations for each set of {*P*, *ν*_*max*_} using contour levels.

This second mode of calculation has some interesting advantages over the first mode. First, a better appreciation of the conformational space of the cycle that is in agreement with the experimental data is obtained. Furthermore, the extent of the contour levels in the pseudorotation plot allow to establish whether the model is under-determined or not. Even when under-determined, such calculation can already indicate those conformations that can be excluded from further investigation.

## Results

### Program features

Only the main features of the program, fully described in the manual available together with the program, are discussed. The GUI is easy to use and handles the input as well as the various operational modes and data output (Figure 1). Two modes of calculation for fitting five-membered ring conformations to ^3^*J*_*HH *_scalar coupling data obtained at up to five temperatures can be selected. Both the coupling data and additional parameters required by equations 1 and 6 are defined interactively, using a simplified representation of the cycle (Figure 1, upper panel). Both the atom types in the cycle and the substituents can be defined interactively. The GUI includes an 'electronegativity editor' (Figure 1, bottom panel) which provides a convenient graphical aid to set up the group-electronegativities (*λ*_*i*_) for many common substituents as required by equation 4.

Also, the program is tolerant for sparse input data. When only a sum of two or more ^3^*J*_*HH *_scalar couplings can be measured, e.g. due to overlap, this sum can be included in the model instead of the individual couplings. Contrary to the PSEUROT program, a coupling does not necessarily have to be measured at each temperature to be included in the calculation.

Four different parameterizations of equation 4 are implemented in the program. These include the 20-parameter and 12-parameter parameterization by Donders [14] and two slightly different 9-parameter parameterizations [28,29] one of which is used in PSEUROT 6.2. The use of Diez-parameters into the calculations is fully implemented by using equation 6 for the calculation of exocyclic torsion angles out of the puckering parameters. The current version does not include the use of the Barfield-correction [30,31] used in some studies.

Depending on the calculation mode selected, textual or graphical output is provided. As the program is written in Matlab, the latter plots can be easily post-processed to comply with the user's needs. In addition, several parameters involving the construction of the plots can be set manually. These parameters include the resolution of the search grid used for the pseudorotation scan (i.e. the increment in *P *and *ν*_*max*_) and the minimal and maximal contour levels used in the plots. Furthermore, interpolation of these contour plots using cubic spline functions can be selected for obtaining smoothed graphs.

Standard data sets for *β*-D-ribose and *β*-D-deoxyribose as well as input data of **1 **and **2 **are available with the distribution of the software.

### Comparison to PSEUROT

**Figure 2:**
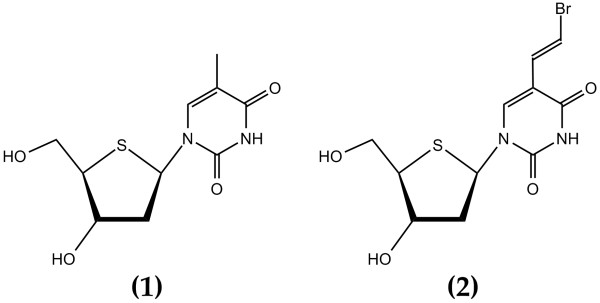
Covalent structure of compounds **1 **and **2**.

To test the performance of the computational core and the input/output handling using the GUI and compare the results with respect to PSEUROT (version 6.2), a set of two 4'-thio-2'-deoxynucleoside analogs [32] was used. For both molecules, depicted in Figure 2, literature [32] provides a full set of five coupling constants measured at five different temperatures as well as Diez-parameters for each endocyclic bond. For both compounds, two conformations were fit to the experimental data using our Matlab GUI and PSEUROT 6.2. The most recent parameterization of equation 4 was used. Details of these optimal fittings, as well as a third fit, taken from literature [32] and referred to as *LHK*, using the Haasnoot equation 3 [12] are presented in Table 1. This table includes the optimized pseudorotation parameters for the North and South conformations (*P*_*N*_, *ν*_*N*_, *P*_*S*_, *ν*_*S*_) in degrees and the population of the North-conformer at each temperature for which couplings were provided. Finally, the maximal deviation and RMSD between the fitted couplings and the experimental ones is indicated.

**Table 1:** Optimal puckering coordinates, partition coefficients and residual errors found for compounds 1 and 2 using this Matlab GUI (*ML*), PSEUROT (*PS*) and literature data (*LHK*) [32].

	**1**			**2**		
	ML	PS	LHK	ML	PS	LHK
*P*_*N*_	3.9	3.6	13	1.8	0.1	9
*ν*_*N*_	41.7	42.6	45	42.3	44.0	45
*P*_*S*_	184.3	182.5	177	184.2	182.5	177
*ν*_*S*_	41.9	41.0	43	42.4	40.9	44
%*N*_285*K*_	29	29	25	36	36	33
%*N*_300*K*_	31	31	-	37	37	-
%*N*_313*K*_	32	32	-	38	38	-
%*N*_333*K*_	35	35	-	39	39	-
%*N*_353*K*_	37	37	33	40	40	37
Δ*J*_*max*_	0.14	0.18	0.21	0.18	0.21	0.29
Δ*J*_*rms*_	0.055	0.060	0.14	0.075	0.083	0.12

Numbers are indicated in degrees, percentages and Hz.

Quasi identical results are obtained when comparing the Matlab GUI (*ML*) to PSEUROT (*PS*). Differences between the two programs are most likely introduced by the difference in optimization method and convergence criteria. Looking at the residual deviations with respect to the experimental data, the Matlab GUI finds a slightly better fit. When comparing to the literature data (*LHK*), one can appreciate the influence of different Karplus equations on the fitting. Differences of 10 and 3 degrees are found for *P*_*N *_and *ν*_*N *_respectively. Also slight differences in populations are observed.

**Figure 3:**
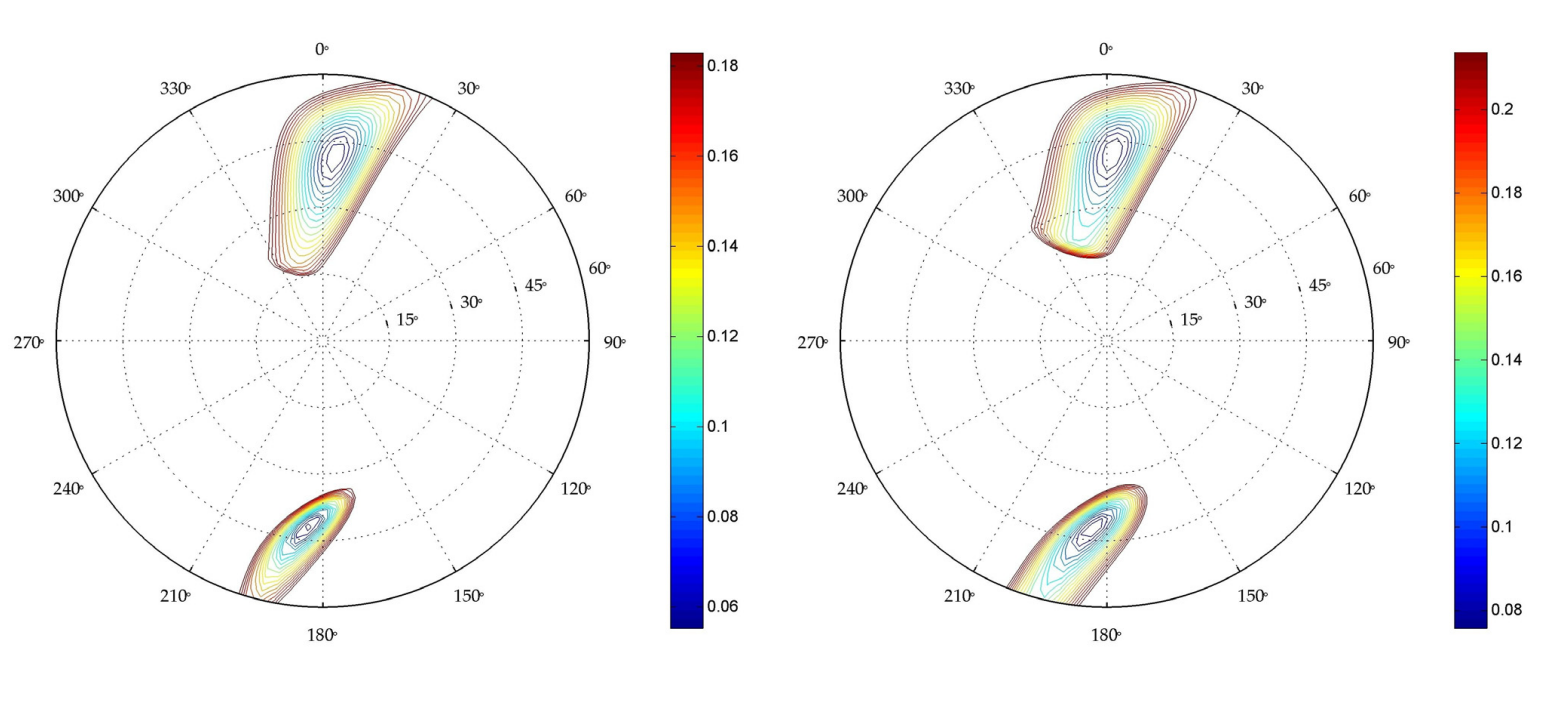
Pseudorotation wheel for compounds **1 **and **2**. Contour lines indicate the total RMSD with the experimental data (Hz). Figures are directly obtained from the GUI. No post-processing has been performed.

**Figure 4:**
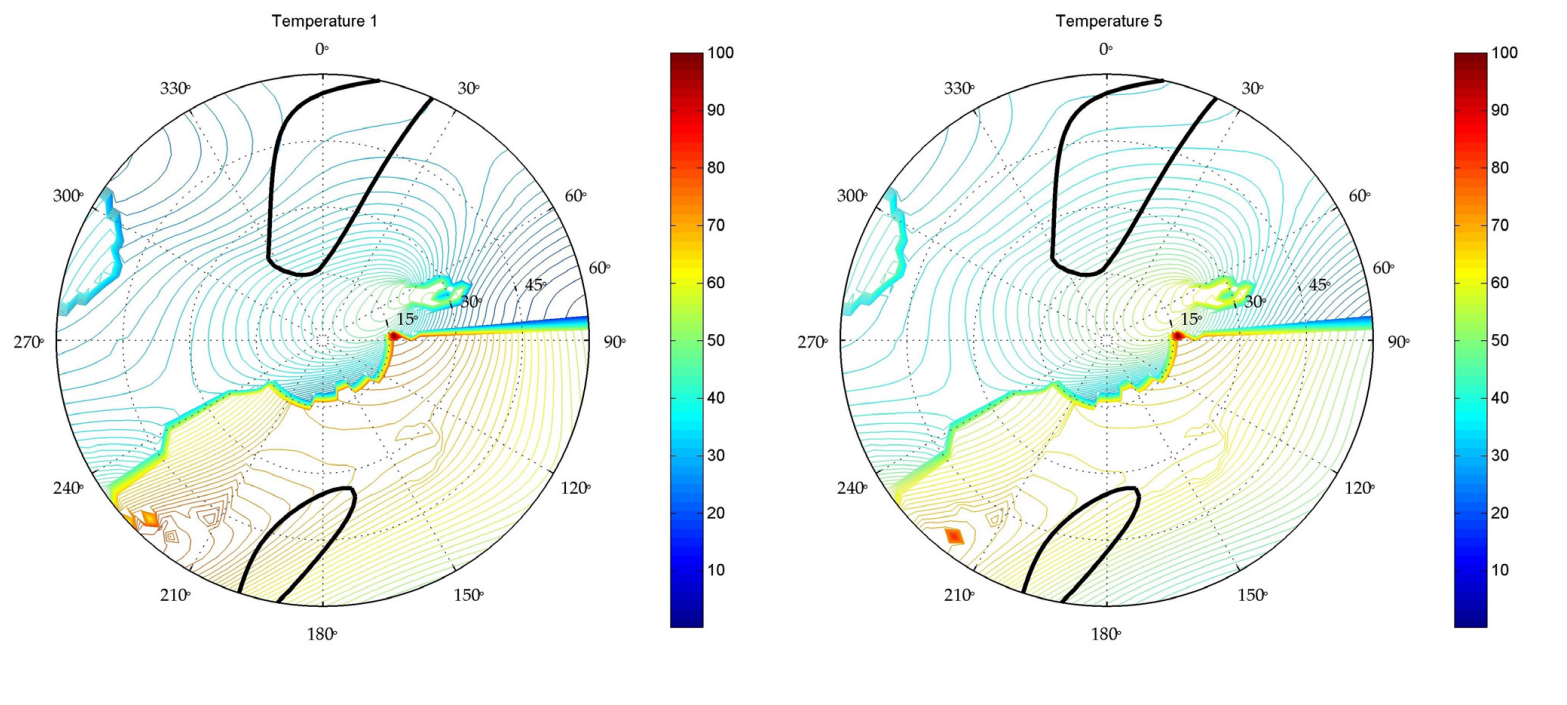
Pseudorotation wheel of partition coefficients of compound **1 **for the two extreme temperatures (285K and 353K). Contour lines indicate the percentage of conformation present in the best fit. The thicker black contour line is the outer contour line of Figure 3. Figures are directly obtained from the GUI. No post-processing has been performed.

In order to get a better appreciation of the precision of these optimal values, the full pseudorotation wheel of **1 **and **2 **has been scanned by the Matlab program resulting in Figures 3 and 4. In Figure 3, contour plots indicate the root-mean-square-deviation between the fitted and the experimental scalar coupling couplings for **1 **(left) and **2 **(right). Looking at the extent of the contour lines, it is clear that the South-conformation is better defined than the North-conformation. Taking into account errors originating from measuring scalar couplings, deficiencies of the model and the Karplus equation, the optimal fit does not necessarily correspond to the real situation. Therefore, interpreting the regions that have a RMSD lower than a certain threshold (e.g. 0.2 Hz), as representative of the cycle's conformation seems more reasonable than taking the optimal fit for granted, especially when comparing different compounds. In addition to Figure 3, Figure 4 can be used to interpret the allowed population-range for each conformation. These graphs represent the population of both conformations of **1 **at 285K (left) and 353K (right). The thick black contour is the highest contour level of Figure 3. Within this black contour, a variety of populations can be found, ranging from 27 to 40 percent of the North-conformation at 285K and from 35 to 49 percent at 353K. Again, this gives a more realistic view of the conformational behavior of compound **1**.

## Conclusion

A matlab program with easy to use graphical user interface for the calculation of five-membered ring conformations has been presented. This program has been made freely available online under a GNU GPL license. The performance of the program was tested on a set of two 4'-thio-2'-deoxynucleoside analogs. It has been shown that identical results can be obtained as in PSEUROT. Furthermore, high-quality graphical output can be generated, facilitating the interpretation of the calculations.

## Availability and requirements

**Project name: **Matlab Pseudorotation GUI

**Project home page: **

**Operating system(s): **Platform independent (tested in WinXP)

**Programming language: **Matlab (tested in release R2007a)

**Other requirements: **Matlab Optimization Toolbox

**License: **GNU GPL

## Authors' contributions

PMSH is the main author of this manuscript. His contributions to this article include the programming of the Matlab program  and GUI, performing extensive tests to ensure its stability and  functionality. Furthermore, the comparison of the obtained results between  the GUI, PSEUROT and literature data were contributions from this author. JCM has provided intellectual, technical and financial  support for the development of the Matlab GUI. Also, he contributed in  testing phase of the GUI.
